# Effects of extremity positioning on radiographic evaluation of femoral tunnel location with digitally reconstructed femoral lateral radiographs after anterior cruciate ligament reconstruction

**DOI:** 10.1186/s12880-015-0093-9

**Published:** 2015-10-24

**Authors:** Parag Suresh Mahajan, Prem Chandra, Nazeer Ahamad, Sheik Akbar Hussein

**Affiliations:** Department of Clinical Imaging, Hamad Medical Corporation, Doha, Qatar; Medical Research Center, Hamad Medical Corporation, Doha, Qatar

**Keywords:** Blumensaat’s line, Anterior cruciate ligament, Alignment, Radiograph, ACL reconstruction, Anatomic single-bundle

## Abstract

**Background:**

Radiographic imaging is a valuable tool in clinical practice for quick anatomical assessment. We aimed to radiographically assess (A) the anterior cruciate ligament (ACL) graft tunnel location after anatomic single-bundle (SB) reconstruction and (B) the effects of extremity positioning on the localization of the orifice of the tunnel in the distal femur in comparison with Blumensaat’s line (BL).

**Methods:**

Three-dimensional computed tomography (3D CT) scan examinations of 22 knees of 22 subjects were evaluated. The 3D CT scan data was used to digitally reconstruct the true lateral radiographs. Graft tunnel location on the distal femoral shaft along the Blumensaat’s line and perpendicular to it were assessed on these radiographs. The femur was digitally rotated to simulate varus, valgus, internal rotation and external rotation in 5-degree increments from 0 to 20-degree. At each incremental rotated position of the femur, position of the ACL graft tunnel was calculated relative to BL and the difference from the true lateral x-ray was estimated.

**Results:**

The position of the tunnel in the distal femur was 30.6 (±4.4) % along BL and 33.1 (±5.4) % perpendicular to BL. Ten and more degree of external, internal, valgus and varus rotations significantly affected the estimates of tunnel position (*P* < 0.05).

**Conclusions:**

Femoral tunnel location can be reliably estimated from lateral radiographs after anatomic SB ACL reconstruction. Although, ten or more degree of rotations can introduce significant inaccuracies in tunnel location estimates, our study suggests that BL is overall reliable for assessing location of the distal femoral tunnel. Level of evidence: Level 2b (Retrospective Cohort Study).

## Background

Although anterior cruciate ligament (ACL) reconstructions are routinely performed to treat ACL injuries, these injuries may have serious consequences [1]. Double-bundle, single-bundle (SB) augmentation and SB techniques are widely practiced to reconstruct the ACL [2]. The double-bundle and SB augmentation techniques are also referred to as anatomic double-bundle and anatomic SB techniques respectively. In anatomic SB ACL reconstruction the femoral tunnel is placed at the site of insertion of the native ACL [3]. Correct tunnel positioning is essential for an optimum clinical outcome in all these techniques [3–8]. Several landmarks and reference points are suggested to aid correct tunnel positioning [9, 10]. Blumensaat’s line (BL) and Bernard et al. technique [9] are commonly adopted to determine the location of the tunnels in the distal femoral shaft on plain x-rays. In a lateral plain x-ray of knee the BL is seen as a projection of the femoral intercondylar notch ceiling or dome [9]. The quadrant technique to estimate the position of femoral tunnel orifices on plain x-rays was first developed by Bernard et al. [9]. In Bernard et al. grid based technique, the ACL graft tunnel position in the distal femoral shaft is computed as a proportion or percentage of the length along the BL from the proximal or anterior femoral cortex to the distal or posterior, and the junction of BL and the distal or posterior cortex is considered as 0 % [9]. This technique is frequently used in both morphological research examinations and examinations reporting the result of ACL reconstruction [11]. For SB reconstruction, 25 % with a standard deviation of ±7 % was suggested as an ideal value [11, 12].

Three-dimensional (3D) imaging techniques, like computerized tomography (CT) and magnetic resonance imaging (MRI) are now commonly used to evaluate ACL injury and progress following ACL reconstruction [11]. Nevertheless two-dimensional (2D) imaging measurements, such as BL on plain x-rays, remain the most common technique for estimating the position of the distal femoral tunnel [10, 13–18], due to its simplicity, easy access, availability, low radiation exposure and low costs. Spatial orientation of the knee joint in the X-ray setup may affect the comparative position of landmarks in a 2D image [11]. Hence, it is envisaged that 2D-radiography is less accurate than 3D-imaging techniques. However a few recent studies suggest similar levels of accuracy with 2D-radiography and 3D-imaging or intraoperative techniques in assessing tunnel/graft position [11, 19–22]. There are very few studies evaluating tunnel placement by radiographs in anatomic single-bundle ACL reconstruction [19, 23]. Hence we (1) radiographically evaluated the influence of femoral rotation on the position of the tunnels in the distal femur relative to BL after anatomic SB ACL reconstruction; and (2) assessed clinical usefulness of radiographic evaluation of femoral tunnel and discuss its role as compared to that of the 3D-imaging techniques. We hypothesized that the radiographic projection of BL changes with femoral rotation, and that extremity malalignment (in comparison to a true lateral x-ray) will induce inaccuracies in estimating the graft tunnel position after SB ACL reconstruction.

## Methods

**Fig. 1:**
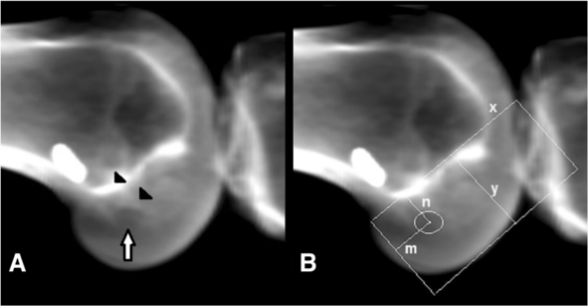
**a** and **b**: Digitally reconstructed radiographs showing distal femoral tunnel (arrowheads) and tunnel aperture (arrow) in 1**a** and Bernard and Hertel grid in 1**b**

All 3D CT scan examinations of knee joints after anatomic single-bundle ACL reconstruction performed in our institute from May 2005 to November 2012 with available 3D CT scan data (22 knees of 22 different subjects) were evaluated after the study was approved by our Institutional Research Ethics Committee (Ethics Committee, Medical Research Center, Hamad Medical Corporation, Doha, Qatar). We received “Waiver of Informed Consent” from the Ethics Committee, as only anonymized images were studied retrospectively. According to van Eck et al. [24], anatomic ACL reconstruction is “the functional restoration of the ACL to its native dimensions, collagen orientation and insertion sites.” Out of the 22 subjects six were females. Subjects ranged in age from 19 to 46 years with average age of 26 years. Time between lesion occurrence and ACL reconstruction ranged from 6 to 13 months with an average time of 8 months. All 22 subjects underwent 3D-CT examination of the operated knee with a 64 slice helical scanner. A three-dimensional volumetric model of femur was created by first segmenting the femur from the adjoining soft tissues and then interposing its CT volume (described as the entire bone volume from external cortical surface to the entire internal tissue) with every single voxel illustrating radiographic density of the local bone. Radiographs were digitally reconstructed by using “Orthopaedics Review” application available under “3D viewer” tools for lower limbs on AW Server two – version aws 2.0–5.5 by GE (General Electric). These reconstructed radiographs (RRs) were generated by ray-tracing through the femoral CT volume model. Spatial orientation inside the virtual radiograph setup that this computer application used was same as that used while obtaining a regular knee x-ray, maintaining a distance of 1 meter between the source and the subject [25]. It rendered an image very similar to an actual x-ray, with prominent radiographic features (such as BL) clearly visible [Fig. 1a and b].

A “true lateral” x-ray was obtained by spatially manipulating the femoral 3D CT model inside the virtual imaging program. The Bernard et al. quadrant technique [9] was employed to estimate the ACL graft tunnel position in relation to the BL. A grid was marked onto the RR using a computer application called ImageJ (ImageJ version 1.47, National Institutes of Health, United States of America). The tunnel outline was observed and its midpoint was estimated. Four lines were drawn to form a grid and four distances (x, y, m and n) were measured along these lines. The “x” is the distance along BL from proximal or anterior aspect of femoral cortex to its distal or posterior aspect. The “y” is the distance along the line which is oriented 90-degree to the BL, from BL to the posterior aspect of the femoral cortex. The “m” is the distance from the proximal or anterior aspect of the femoral cortex to the midpoint of the tunnel along “x”, and the “n” is the distance from the BL to the midpoint of the tunnel along “y”. The midpoint of the ACL graft tunnel was illustrated as a percentage of two lengths along each axis. The percentage of the length along BL was called as “%BL” and computed as m/x. The %BL was measured from the distal or posterior to the proximal or anterior femoral condylar cortex, and 0 % denoted the junction of this line with the distal or posterior femoral condylar cortex. The percentage of the length along the line 90-degree to BL was called as %DP. The %DP was computed as n/y and measured from BL to the inferior cortex of the femoral condyle [Fig. 1a and b], with 0 % at the position on BL.

**Fig. 2:**
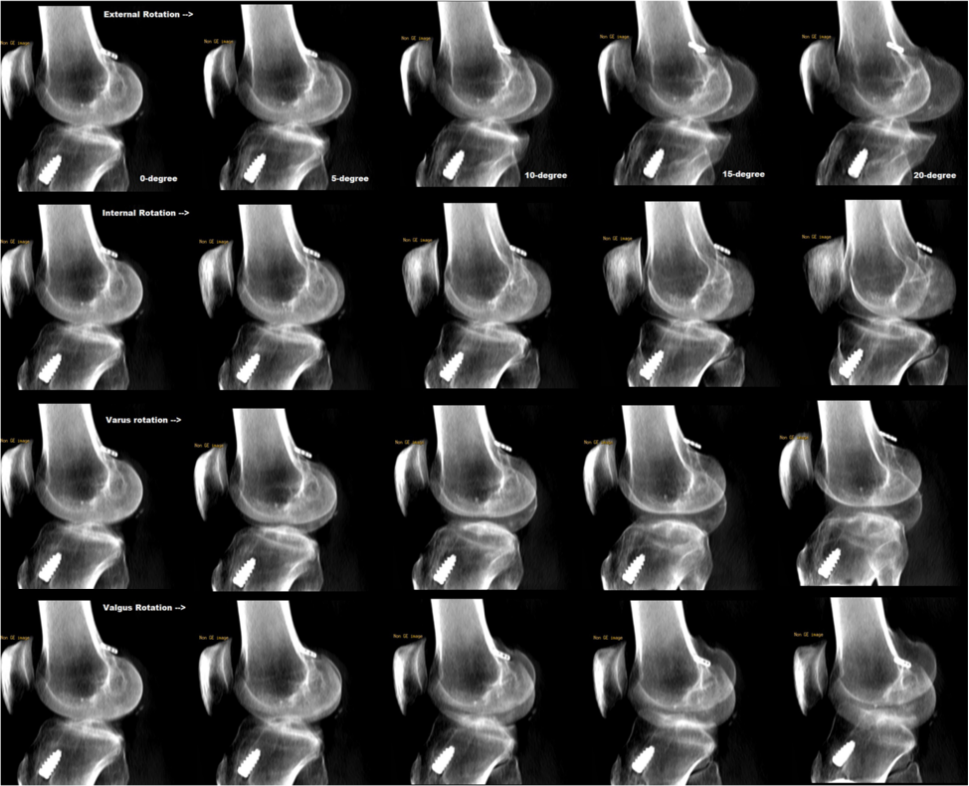
Thumbnails of digitally reconstructed radiographs of a representative case in 0 to 20-degree of external, internal, varus and valgus rotations. 0-degree rotation represents a true lateral radiograph

Subsequently, the femoral 3D CT reconstruction was moved spatially within the setup of virtual radiograph computer program to simulate varus, valgus, internal rotation and external rotation in 5-degree additions from the base level of 0 degree to 20-degree. An RR was produced at each 5-degree augmentation [Fig. 2]. The Bernard et al. technique as explained above was applied to each RR to estimate the ACL graft tunnel position in relation to the BL [11]. All parameters were measured by two radiologists to determine the inter-observer reliability. Final statistical analysis was performed after averaging the measurements by the two radiologists.

### Statistical analysis

We computed ICC to assess inter-observer variability for %BL and %DP positions of ACL graft tunnel orifices. Mean, range and standard deviation of all ACL graft tunnel orifice positions of %BL and %DP were computed. We used repeated-measures ANOVA to determine if %BL and %DP locations of the ACL graft tunnel orifices changed with each 5-degree augmentation of varus, valgus, internal and external femoral rotation. When the repeated-measures ANOVA was significant (*p* < 0.05), we performed post hoc tests with Wilcoxon matched-pairs signed ranks test. In this test, rotation smaller than 10-degree was compared to rotation greater than 10-degree to determine if a 10-degree change in extremity alignment significantly altered ACL graft tunnel orifice positions of %BL and %DP. Pictorial presentations of the key results were made using appropriate statistical graphs. A two-sided *P* value <0.05 was considered to be statistically significant. All Statistical analyses were done using statistical packages SPSS 19.0 (SPSS Chicago, IL).

## Results

The inter-observer variability and standard error of measurement (SEM) along with 95 % confidence interval (CI) of the measured ACL graft tunnel orifice positions in relation to the BL are presented in Table 1. Very good inter-observer agreement was observed under this study. Intra-class correlation coefficient for x (distance from posterior to anterior cortex along BL), y (distance from BL to femoral cortex), m (distance of tunnel from posterior femoral cortex to center of tunnel along BL), n (distance of tunnel from BL to center of tunnel perpendicular to BL), %BL (% BL of tunnel) and %DP (% DP of tunnel) values were 0.971 (95 % CI: 0.962–0.980), 0.930 (95 % CI: 0.906–0.950), 0.887 (95 % CI: 0.848–0.919), 0.758 (95 % CI: 0.675–0.827), 0.829 (95 % CI: 0.770–0.878) and 0.758 (95 % CI: 0.675–0.827) respectively.

**Table 1:** Inter-observer variability and standard error of measurement of quadrant methods for evaluation of tunnel aperture orifice after anatomic single-bundle anterior cruciate ligament reconstruction

	ICC^a^	CI^b^	SEM^c^
x - Distance from posterior to anterior cortex along BL	0.971	0.962, 0.980	3.06
y - Distance from BL to femoral cortex	0.930	0.906, 0.950	2.46
m - Distance of tunnel from posterior femoral cortex to center of tunnel along BL	0.887	0.848, 0.919	3.73
n - Distance of tunnel from BL to center of tunnel perpendicular to BL	0.758	0.675, 0.827	2.80
%BL of tunnel	0.829	0.770, 0.878	1.69
%DP of tunnel	0.758	0.675, 0.827	2.36

*BL* Blumensaat’s line

^a^Intra-class correlation coefficient

^b^95 % confidence interval of the intra-class correlation coefficient

^c^Standard error of measurement

**Fig. 3:**
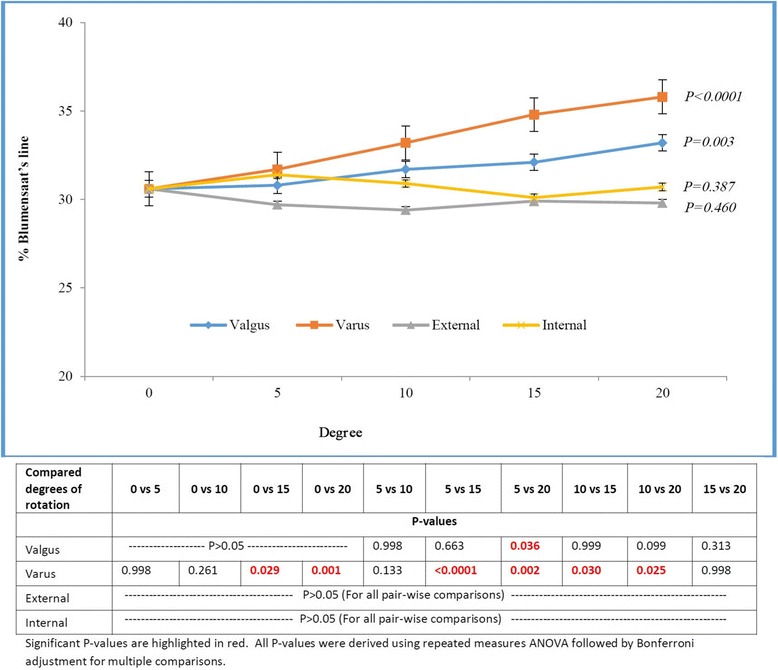
Femoral ACL tunnel aperture location expressed as percentage along Blumensaat’s line in relation to degree of rotation for various rotation types. For valgus rotation, *P* values were significant (*P* < 0.05) only for compared degrees - ‘5 vs 20’. For varus rotation, *P* values were significant (*P* < 0.05) only for compared degrees - ‘0 vs 15’, ‘0 vs 20’, ‘5 vs 15’, ‘5 vs 20’, ‘10 vs 15’, and ‘10 vs 20’. For external rotation, *P* values were not significant (*P* > 0.05) for all pair-wise comparisons. For internal rotation, *P* values were not significant (*P* > 0.05) for all pair-wise comparisons

**Fig. 4:**
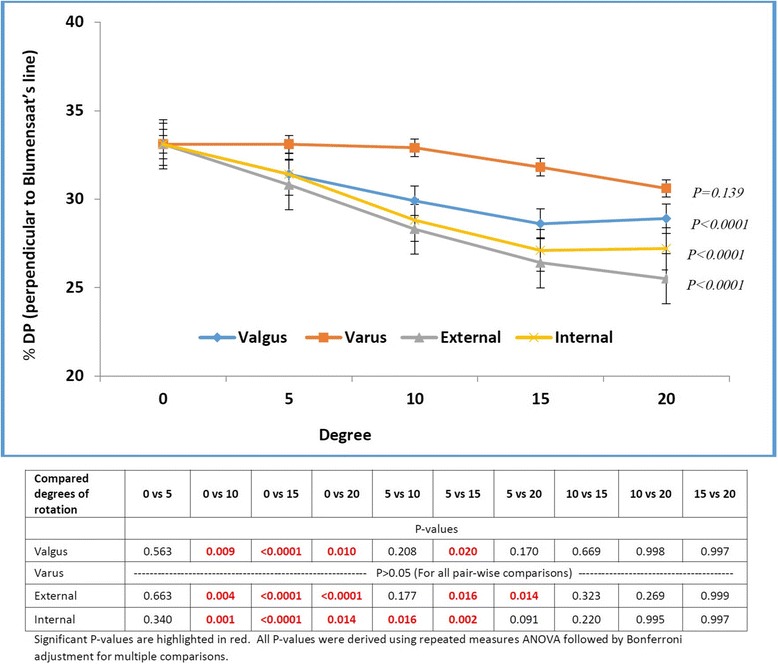
Femoral ACL tunnel aperture location expressed as percentage along the line perpendicular to the Blumensaat’s line (%DP) in relation to degree of rotation for various rotation types. For valgus rotation, *P* values were significant (*P* < 0.05) only for compared degrees - ‘0 vs 10’, ‘0 vs 15’, ‘0 vs 20’, and ‘5 vs 15’. For varus rotation, *P* values were not significant (*P* > 0.05) for all pair-wise comparisons. For external rotation, *P* values were significant (*P* < 0.05) only for compared degrees - ‘0 vs 10’, ‘0 vs 15’, ‘0 vs 20’, ‘5 vs 15’, and ‘5 vs 20’. For internal rotation, *P* values were significant (*P* < 0.05) only for compared degrees – ‘0 vs 10’, ‘0 vs 15’, ‘0 vs 20’, ‘5 vs 10’, and ‘5 vs 15’

The mean ACL graft tunnel orifice positions for all 5-degree increases for all rotation types (external, internal, valgus and varus) are presented in Table 2. In our study the ACL graft tunnel position was 30.6 (±4.4) %BL and 33.1 (±5.4) %DP. The %BL measurements of tunnel position did not show any significant change for 0 to 20 degree of external, internal and valgus rotations (*P* > 0.05) [Fig. 3]. Fifteen and more degree of varus rotation resulted in a significant different position of the tunnel (only for %BL measurements) along Blumensaat’s line (*P* = <0.0001) [Fig. 3]. The %DP measurements of tunnel position did not show any significant change for 0 to 20 degree of varus rotation (*P* > 0.05) [Fig. 4]. Ten and more degree of external, internal and valgus rotations resulted in a significant different position of the tunnel (only for %DP measurements) along Blumensaat’s line (*P* < 0.0001) [Fig. 4]. We did not find any significant variability in the position of tunnel for each 5 degree increase of rotation amongst the 22 subjects in our study related to skeletal morphological differences.

**Table 2:** Tunnel positions relative to Blumensaat’s line

Rotation Type	Lateral	Valgus				Varus			
Degree	0	5	10	15	20	5	10	15	20
%BL	30.6 ± 4.3	30.8 ± 4.1	31.7 ± 4.5	32.1 ± 5.1	33.2 ± 5.5	31.7 ± 4.5	33.2 ± 4.6	34.8 ± 4.7	35.8 ± 5.1
%DP	33.1 ± 5.4	31.4 ± 5.7	29.9 ± 5.1	28.6 ± 4.2	28.9 ± 5.3	33.1 ± 6.0	32.9 ± 5.8	31.8 ± 5.7	30.6 ± 7.5
Rotation Type	Lateral	External				Internal			
Degree	0	5	10	15	20	5	10	15	20
%BL	30.6 ± 4.3	29.7 ± 4.2	29.4 ± 4.3	29.9 ± 3.4	29.8 ± 4.1	31.4 ± 4.7	30.9 ± 5.2	30.1 ± 5.2	30.7 ± 5.3
%DP	33.1 ± 5.4	30.8 ± 6.5	28.3 ± 5.0	26.4 ± 4.2	25.5 ± 5.7	31.4 ± 5.4	28.8 ± 5.8	27.1 ± 5.3	27.2 ± 7.9

*BL* Blumensaat’s line, *DP* perpendicular to Blumensaat’s line

## Discussion

The most important finding of our study is that the BL is quite reliable in evaluating graft tunnel position after SB ACL reconstruction. However ten and more degree of external, internal, valgus and varus rotations significantly affected estimates of radiographic tunnel position. Less than 10-degree of external, internal, valgus and varus rotations did not have any significant effect on estimates of tunnel position. Our study demonstrates that the locations of tunnels after SB ACL reconstructions can be reliably estimated on true lateral x-rays, thus affirming our primary objective. In case of SB reconstructions, ten and more degree of external, internal, valgus and varus rotations significantly affected estimates of tunnel position. Less than 10-degree of external, internal, valgus and varus rotations did not have any significant effect on estimates of tunnel position. This is contrary to our hypothesis. Similar impact of rotations on both the tunnel orifice position and the BL for less than 10-degree rotations is the likely explanation for it. Expressing the ACL graft tunnel position in percentage terms (%BL along the BL and %DP along the line 90-degree to the BL), may compensate these distortions.

Skeletal morphological differences amongst the subjects may also be a conducive factor [11, 26]. We however did not find any significant variability in the position of tunnel for each 5 degree increase of rotation amongst the 22 subjects in our study related to skeletal morphological differences.

The quadrant method by Bernard et al. is a 2D technique. Despite the invention of newer 3D imaging methods, this 2D technique for placement of ACL graft tunnel is extensively used as it is easily available, cost-effective and simple to perform. The BL is just a radiographic projection of an anatomical structure (intercondylar notch ceiling) and not a genuine anatomical structure [11]. This is the reason, why it could be influenced by extremity malalignment and by patient’s skeletal anatomy. Thus, when the femur is rotated in relation to the source of the X-ray, BL may not depict the cranial most aspect of the intercondylar notch ceiling.

From a clinical point of view, our study indicates that the BL is quite reliable in evaluating graft tunnel position after ACL reconstruction. However, if the lateral x-ray is taken with inappropriate extremity positioning, significant inaccuracies may occur. Therefore, we would advise implementation of methods that consider the individual subject’s anatomy and standardize extremity positioning. If a strict limb positioning algorithm is followed to restrict limb malpositioning within 5-degree of any rotation, the radiographic evaluation can be clinically as useful as 3D-imaging techniques. Intraoperative fluoroscopy may be used for misplacement prevention. 3D CT scan or MRI scanning may be utilized where available to yield higher accuracy [11, 27, 28].

van Eck et al. [11] were the first to study the effects of limb alignment on ACL graft tunnel positions estimated from plain radiographs. They studied these effects in double-bundle and nonanatomic single bundle reconstructions. They found that, after double-bundle reconstruction, valgus rotation greater than 10° significantly affected the assessment of tunnel position (*P* = 0.043) and after nonanatomic single-bundle reconstruction, internal rotation of more than 10° significantly affected the assessment of tunnel position (*P* = 0.043). We studied the effects of limb alignment on tunnel positions in anatomic single-bundle reconstructions, which to our knowledge, were not evaluated before. Lee et al. [19] evaluated femoral tunnel positioning using 3D CT and radiographs after single-bundle ACL reconstruction with modified transtibial technique and found these to be very useful. They concluded that, their modified transtibial technique is anticipated to provide more anatomical placement of the femoral tunnel than the former traditional transtibial techniques. Borbas et al. [20] used radiodense ligament markers for radiographic evaluation of ACL reconstruction. They found the application of radiodense ACL graft markers as straight-forward, non-expensive and potentially useful to identify the position of the graft and for diagnosis of graft failure on antero-posterior radiographs. On lateral radiographs, however, marker distances were highly variable and did not correlate with clinical ACL graft failure. Pietrini et al. [21] studied radiographic landmarks for tunnel positioning in double-bundle ACL reconstructions. This study defined the radiographic locations of the femoral and tibial bundle attachment sites of the native ACL and also defined a reliable and transferable protocol for identifying these sites on radiographs in relation to surrounding landmarks and digitally projected reference lines. Jenny et al. [22] compared the intraoperative navigated measurements of the location of the tibial and femoral tunnels during arthroscopic-assisted ACL reconstruction to the postoperative measurements performed on standard plain antero-posterior and lateral radiographs. They found significant correlation between intraoperative navigated and postoperative radiographic measurements only at the femur and good agreement between all navigated and radiographic measurements.

Performing the measurements on computer generated x-rays (RRs), rather than actual x-rays is one of the limitations of our study. Though the appearance and quality of the RRs is slightly different from regular x-rays, use of computer generated images provided precise definition of imitated rotation and true lateral position with a resolution of 1 degree accuracy. Moreover, our technique had very good inter-observer agreement. Comparatively smaller number of included subjects is another limitation of our study. More such studies with larger number of subjects may be needed to confirm the findings of our study.

## Conclusions

Femoral tunnel location can be estimated from lateral radiographs after anatomic SB ACL reconstruction. Although, ten and more degree of external, internal, valgus and varus rotations can introduce significant inaccuracies in tunnel location estimates, our study suggests that BL is overall reliable for assessing location of the distal femoral tunnel and more so if a strict limb positioning algorithm is followed to restrict limb malpositioning within 10-degree of any rotation.

## Abbreviations

ACL: Anterior cruciate ligament
SB: Single-bundle
BL: Blumensaat’s line
3D CT: Three-dimensional computed tomography
3D: Three-dimensional
CT: Computerized tomography
MRI: Magnetic resonance imaging
2D: Two-dimensional
GE: General electric
RRs: Reconstructed radiographs
ICC: Intra-class correlation coefficient
%BL: The percentage of the length along BL
DP: Line perpendicular to Blumensaat’s line
%DP: The percentage of the length along the line 90-degree to BL
ANOVA: Analysis of variance
SEM: Standard error of measurement
CI: Confidence interval
